# Multiple-choice quizzes improve memory for misinformation debunks, but do not reduce belief in misinformation

**DOI:** 10.1186/s41235-023-00488-9

**Published:** 2023-06-06

**Authors:** Jessica R. Collier, Raunak M. Pillai, Lisa K. Fazio

**Affiliations:** 1 Center for Media Engagement, University of Texas at Austin, Austin, USA; 2 Department of Psychology and Human Development, Vanderbilt University, Nashville, USA; 3 Department of Communication, Mississippi State University, P.O. Box PF, Mississippi State, MS 39762 USA

**Keywords:** Quizzes, Fact-checking, Retrieval practice, Misinformation, Debunk

## Abstract

Fact-checkers want people to both read and remember their misinformation debunks. Retrieval practice is one way to increase memory, thus multiple-choice quizzes may be a useful tool for fact-checkers. We tested whether exposure to quizzes improved people’s accuracy ratings for fact-checked claims and their memory for specific information within a fact check. Across three experiments, 1551 US-based online participants viewed fact checks (either health- or politics-related) with or without a quiz. Overall, the fact checks were effective, and participants were more accurate in rating the claims after exposure. In addition, quizzes improved participants’ memory for the details of the fact checks, even 1 week later. However, that increased memory did not lead to more accurate beliefs. Participants’ accuracy ratings were similar in the quiz and no-quiz conditions. Multiple-choice quizzes can be a useful tool for increasing memory, but there is a disconnect between memory and belief.

## Introduction

Fact-checking organizations have proliferated recently as the opportunity for exposure to false or misleading information has increased. Fact-checking refers to “the practice of systematically publishing assessments of the validity of claims made by public officials and institutions with an explicit attempt to identify whether a claim is factual” (Walter et al., 2020, p. 351). These published assessments, or articles, constitute fact checks. The growth of fact-checking comes at a time when communicating the validity of claims has important ramifications from support for vaccines to belief in the legitimacy of elections (Painter & Fernandes, 2022; Zhang et al., 2021). Although fact-checking is not the only means for combating misinformation, its benefits are well-documented (Dunn et al., 2015; Fridkin et al., 2015; Gottfried et al., 2013; Weeks & Garrett, 2014; Wood & Porter, 2019). For fact checks to be effective in the most basic sense, two things must happen. First, fact checks must stand out in a sea of online content, garnering the attention of readers. Second, once a fact check has been read, it must be retained in the reader’s memory and affect the reader’s beliefs. While there may be other variables specific to an individual that influence whether the fact check is appropriately received (e.g., trust in the author), attention and memory are key. One low-cost tool that has been shown to increase engagement with online news content is multiple-choice quizzing. Here, we examine whether such quizzes may be useful to fact-checkers seeking to improve reader’s memory and beliefs.

Online quizzes have gained recent attention as a tool for news producers to reach readers (The Learning Network, 2021; Wojdynski, 2019). A wide range of outlets publish knowledge quizzes including *USA Today*, CNN, and *Wall Street Journal*. *The New York Times* publishes The News Quiz weekly, which quizzes readers on details from the week’s biggest news stories (e.g., “The Supreme Court this week heard a case about whether Colorado law could require a business owner, who works in what industry, to serve same-sex couples?”, “Russia released Brittney Griner, the American basketball star, from a penal colony under what condition?”; News Quiz, 2022).^1^

Recent research has highlighted that such quizzes can increase the time spent engaging with online news articles (Scacco et al., 2016), as they offer an additional means of interacting with news content. While some evidence suggests people do voluntarily read fact checks (Mattes & Redlawsk, 2020), quizzes might entice people to consume fact checks to a greater extent. This effect is important for fact-checking institutions, as time spent on site is a key metric for the survival of online content producers (Hindman, 2015). In addition, by increasing interactivity with online news, quizzes can increase readers’ interest in political news, political engagement, and overall knowledge of public affairs (Masullo et al., 2020; Scacco et al., 2016). In sum, quizzes present a practical option for fact-checkers aiming to encourage readers to engage more deeply with fact checks.

Beyond their engaging quality, quizzes also present an opportunity to increase readers’ memory for accurate information. A large literature demonstrates that the simple act of retrieving information from memory enhances retention of the material, even when compared with restudying the same material (see Roediger & Butler, 2011; Rowland, 2014 for reviews). This testing effect occurs with multiple types of questions and activities, including multiple-choice quizzes (e.g., Roediger & Marsh, 2005; see Marsh et al., 2007 for a review). The memorial benefits of multiple-choice quizzes have been observed with naturalistic materials (e.g., prose passages on science and history from the SAT II; Marsh et al., 2009) and with complex, higher-order quiz content (e.g., summaries of key themes from a passage; Agarwal, 2019). These positive effects on memory occur even several weeks after the initial test (Carpenter et al., 2008, 2009). While much of the existing research has focused on educational settings and materials, these findings suggest that multiple-choice quizzes may also enhance memory for details in fact checks.

If multiple-choice quizzes do improve memory for fact check details, a key practical question is where they may most effectively be placed: before or after reading the article. Theoretically, quizzes in either position should enhance memory. As mentioned above, placing quizzes after an article allows readers to retrieve recently studied information from memory. Retrieving information may strengthen memory by serving as a form of processing that aligns with how learners will eventually be evaluated, allowing learners to exert effort during initial processing, or creating an elaborate memory representation that is easier to access later (see Karpicke, 2017; McDermott, 2021 for reviews). Regardless of the exact mechanism, it is clear that retrieving studied information enhances memory. However, quizzes may also be effective when placed before an article. Quizzes offer an opportunity for test-potentiated learning, in which retrieval practice enhances the encoding of information in a subsequent study trial (e.g., Arnold & McDermott, 2013; Little & Bjork, 2016; Richland et al., 2009). In one recent study, multiple-choice quizzes presented before a text passage were just as effective at enhancing memory as quizzes after the text passage, if not more so (Pan & Sana, 2021).

Another important question is whether the memorial benefits of quizzes are limited to the quizzed content. Fact-checking articles introduce readers to many details, and it would be impractical to quiz readers on each one. Thus, it is important to examine whether quizzing readers on details from the article improves memory for just those few details, or if the memorial benefits extend to other, non-quizzed details as well. Several studies have found that tests also boost memory for non-tested study material that is conceptually related to the tested content (e.g., Chan, 2010; Chan et al., 2006; Little et al., 2012). This boost may occur because as people try to answer the questions, they search their memory for other, related information, enhancing memory for that content. However, this benefit is often smaller than the increase in memory for the tested content itself (e.g., Chan et al., 2006; see Hamaker, 1986 for a review). Relatedly, for tests placed before studied information (i.e., pretests), the benefits of testing are typically limited to the studied information (e.g., James & Storm, 2019; Richland et al., 2009; Toftness et al., 2018). In sum, quizzes may, in some circumstances, boost memory even for non-quizzed content from the article.

Finally, while our focus thus far has been on how quizzes affect memory for the passage, a key outcome of interest for fact-checkers is readers’ beliefs regarding the truth of fact-checked claims. It is important that fact checks improve the accuracy of peoples’ beliefs. Existing research suggests that belief change is best supported when fact-checking messages (e.g., articles, social media posts) provide extra details that explain why a claim is false rather than just restating a claim and providing a true or false label (Chan et al., 2017; Ecker et al., 2020b; Swire et al., 2017). One explanation is that these details can be later retrieved from memory and used as cues to evaluate a claim as false (Brashier & Marsh, 2020). Consistent with this account, fact checks become less effective over time, as memory for the corrective information fades (Swire-Thompson et al., 2023). Retrieval practice is likely to boost memory for details that may indicate a claim is false. Thus, by increasing memory for details of the article, retrieval practice may also increase belief change.

### Current studies

In the current set of preregistered online experiments, we examine whether exposure to a quiz accompanying a fact check improves peoples’ belief accuracy and memory for information in the fact check. In Experiment 1, participants read a fact check refuting health-based misinformation and either did not take a two-item multiple-choice quiz or took the quiz before or after reading the article. They then answered a series of memory and belief questions either immediately or 1 week later. In addition to addressing our main question, Experiment 1 also allowed us to examine whether quizzes were more effective when placed before or after the article, and whether quizzes increase memory for the article as a whole or only for the tested details. Experiment 2 replicates Experiment 1 but removes the quiz-before condition and uses questions that are more directly related to the false claim to assess whether the content of the quiz questions influences their efficacy. Finally, in Experiment 3, we incorporate an additional context by switching to fact checks refuting political-based misinformation. Participants read multiple true and false fact checks. We also added an additional measure of belief, and a control condition exposed to no fact checks. Across all three experiments, we predicted that multiple-choice quizzes would increase participants’ memory for information within the fact check and would decrease their belief in false claims.

## Experiment 1

### Method

Experiment 1 explores how including a quiz before or after reading a fact check influences individuals’ accuracy rating for the false claim and their memory for information in the article. To address our hypotheses, we conducted an online survey experiment administered through Qualtrics. We used a 3 (quiz condition: quiz before, quiz after, no quiz) × 2 (article condition: DNA, marijuana) × 2 (delay: no delay, 1-week delay) between-subjects design. In addition, for subjects who received a quiz, the material on the final cued-recall test was either previously quizzed or not quizzed (manipulated within-subjects). Data were collected from February 4 to 19, 2020.

#### Participants

Participants (*N* = 910) were recruited through Amazon mTurk using the CloudResearch platform (Litman et al., 2017). Our preregistered sample size (*N* = 900) was chosen in order to have 75 participants assigned to each between-subjects condition. The additional 10 participants were due to automatic oversampling in mTurk to account for participants who might not finish the experiment. US residents aged 18 years or older were eligible to participate. Samples from such crowdsourcing platforms are more representative than convenience samples (Berinsky et al., 2012), and responses from CloudResearch-approved participants are of higher quality than responses from general mTurkers (Eyal et al., 2021; Litman et al., 2017). Participants were compensated $1.21 for their participation in the initial survey and $0.25 for their participation in the second survey 1 week later (if they were in the delayed test condition). As preregistered, participants assigned to the delayed test condition who did not participate in the second session (*n* = 83) were excluded. In addition, participants who failed to answer all four cued-recall questions (*n* = 12) were excluded from the analyses. This exclusion criteria left 815 participants in the final sample. A sensitivity analysis in G*Power (Faul et al., 2007) indicated that this final sample size had 80% power to detect a between-subjects main effect of quiz condition (quiz before, quiz after, no quiz) on accuracy ratings of at least *f* = 0.11.

The demographic makeup of the sample was primarily White (76.5%), and 52.2% of the participants were male. Average age was 40.3 years old (*SD* = 11.4). The average time to complete the self-paced survey was 8 min and 56 s on the initial survey and 4 min and 22 s on the delayed survey.

#### Materials

To minimize the influence of topic differences, we tested the efficacy of the quiz intervention for two different articles pertaining to health misinformation. The first was an article about DNA titled, “Do Women Retain DNA from Every Man They Have Slept With?” and the second was an article about marijuana titled, “Did a New Study Show that Marijuana Leads to a Complete Remission of Crohn’s Disease?” Both articles were fact checks from Snopes that rated the claims as entirely false.

For each article, we created four multiple-choice questions about details within the article (e.g., What is Crohn’s disease? (A) a balloon-like bulge in the aorta, (B) A bleeding disorder where the blood does not clot, (C) Swelling of the brain and spinal cord, (D) An inflammatory bowel disease). We split these four questions into two sets of two questions each to counterbalance question exposure such that for those participants in the quiz conditions, the post-treatment cued-recall questions would reflect a mix of quizzed and not quizzed material.

On the final test, each multiple-choice question was turned into a cued-recall question by removing the answer choices (e.g., What is Crohn’s disease?). Participants answered all four cued-recall questions and were instructed to respond “don’t know” if they didn’t know the answer. Again, for participants who saw the multiple-choice quiz earlier, this meant that they answered two questions corresponding to previously quizzed content and two questions about non-quizzed content. To measure participants’ belief in the false claim, they were presented with the debunked statement of the fact check to which they were assigned and were asked to rate its accuracy. For the DNA article, this was, “Women retain DNA from every man they have ever slept with,” and for the marijuana article, “Marijuana leads to a complete remission of Crohn’s disease.” Responses were measured on an 11-point scale from *very inaccurate* (0) to *very accurate* (10). As both claims were entirely false, we expected participant accuracy ratings to decrease in the quizzed conditions.

All materials are available on our OSF site https://osf.io/rfchq/?view_only=0185e2f8c3a343cea3bff597dde2f979.

#### Procedure

Participants were randomly assigned to read one of two articles (DNA, marijuana) about a piece of health misinformation. Participants were instructed to “Please read the article as you would read a typical article on the internet.” Depending on their condition, participants received a brief, two-question multiple-choice quiz about the information in the fact check either before reading the article, after reading the article, or they received no quiz at all. After each question, participants received feedback about the correct answer. For example, if participants answered correctly, the feedback would say “CORRECT” followed by the answer choice they had chosen. If participants answered incorrectly, the feedback would say “INCORRECT” followed by the correct answer choice.

After reading the article and answering any quiz questions, half of participants were asked to immediately rate the accuracy of the false claim from the article and answer four cued-recall questions about the content of the fact check. Participants were instructed to answer each cued-recall question in 1–2 sentences. For participants who took the earlier quiz, two of the questions were repeated from the multiple-choice quiz and two were new. Participants then completed a basic demographic questionnaire and were compensated for their participation.

Participants in the delayed test condition were dismissed immediately after reading the article and completing any quiz questions. One week later, they were invited to the second survey. Participants were first prompted with, “Last week, we asked you to read an article from a fact-checking organization. We would now like to ask you a few questions about the information in that article.” They were then asked to rate the accuracy of the claim, answer the four cued-recall questions, and complete the demographic survey.

### Results

All data are available at the online supplement, along with preregistration of our analyses, hypotheses, and sample size: https://osf.io/rfchq/?view_only=0185e2f8c3a343cea3bff597dde2f979.

#### Multiple-choice quizzes

First, we present participants’ accuracy on the multiple-choice quiz. As shown in Table 1, participants were above chance on the quiz, although they were more accurate when the quiz was placed after the article. We conducted a 2 (quiz: before, after) × 2 (article: DNA, marijuana) ANOVA on the proportion of multiple-choice quiz questions answered correctly. There was a significant main effect for quiz condition (*F*(1, 542) = 59.2, *p* < 0.001, *η*_*p*_^2^ = 0.10) such that participants were more accurate after reading the article. We also observed a significant main effect of article (*F*(1, 542) = 19.8, *p* < 0.001, *η*_*p*_^2^ = 0.04) with higher accuracy for the DNA article. Finally, there was a significant interaction between the quiz and article conditions (*F*(1, 542) = 21.9, *p* < 0.001, *η*_*p*_^2^ = 0.04). In the quiz-after condition, participants answered more quiz questions correctly when they had read the DNA article compared to when they had read the marijuana article.

**Table 1:** Proportion correct on the initial multiple-choice test split by article and quiz timing

Article	Before article	After article	*M*
DNA	0.48 (.32)	0.78 (.33)	0.64
Marijuana	0.49 (.20)	0.56 (.27)	0.52
*M*	0.48	0.67	

Standard deviations are in parentheses

#### Cued-recall questions

Next, we examined participants’ responses to the cued-recall questions. Participants received four open-ended questions about the content from each fact check. Two independent research assistants coded these responses to identify whether they were *correct* or *incorrect*. Answers were coded as correct if they included the entire correct response on the corresponding multiple-choice question. For example, if participants answered the question, “What is Crohn’s disease?” by saying “an inflammatory bowel disease,” their response would be coded as correct. In addition, answers that restated the idea from the correct response but rephrased the response were counted as correct (e.g., “disease caused by inflammation of the intestines”). Partially correct responses (“an inflammatory disease,” or “a bowel disease”), incorrect responses, and don’t know responses were scored as incorrect. Krippendorff’s alpha for this measure was 0.89. All discrepancies were resolved by a co-first author.

**Fig. 1:**
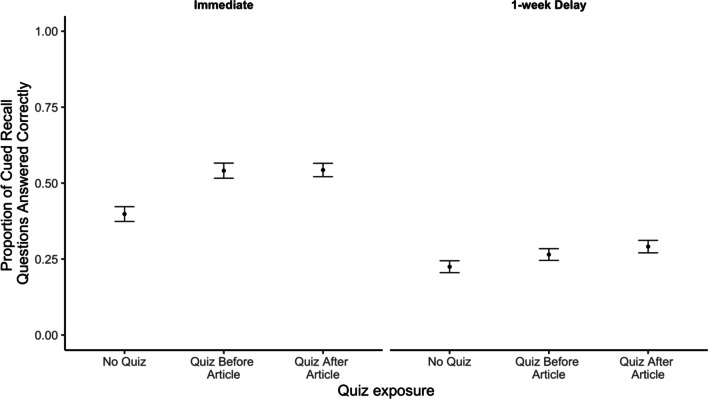
Proportion of cued-recall questions answered correctly by quiz condition and test delay. *Note* Error bars reflect standard errors of the mean

Our key question was whether quizzing would affect participants’ memory for details from the article. We found that it did. Participants who received a quiz accompanying their fact check answered more questions correctly compared to participants who did not receive a quiz (Fig. 1).

We conducted a preregistered 3 (quiz: none, before, after) × 2 (article: DNA, marijuana) × 2 (delay: immediate, delayed) ANOVA on the proportion of correct responses on the cued-recall questions.^2^ We observed a significant main effect of quizzing (*F*(2, 803) = 13.49, *p* < 0.001, *η*_*p*_^2^ = 0.03) such that participants answered more questions correctly when they were quizzed before (*M* = 0.40) or after (*M* = 0.44) reading a fact check, as compared to participants who were not quizzed (*M* = 0.32). Post hoc Tukey tests found a significant difference between the no-quiz and quiz-before conditions (*t*(424) = 3.73, p < 0.001, *d* = 0.33) and the no-quiz and quiz-after conditions (*t*(424) = 4.82, *p* < 0.001, *d* = 0.43). There was also a significant effect of article condition (*F*(1, 803) = 132.09, *p* < 0.001, *η*_*p*_^2^ = 0.14) such that participants answered a greater proportion of questions correctly when they read the marijuana (*M* = 0.49) compared to the DNA (*M* = 0.27) article. As predicted, we also observed a significant main effect of delay (*F*(1, 803) = 151.35, *p* < 0.001, *η*_*p*_^2^ = 0.16) where participants who answered questions immediately after reading the fact check (*M* = 0.50) were more accurate than participants who answered 1 week later (*M* = 0.26). Finally, we observed a significant interaction of article and delay (*F*(1, 803) = 14.02, *p* < 0.001, *η*_*p*_^2^ = 0.02) such that the effect of delay was larger for the marijuana article. The interactions between quiz condition and delay (*F*(2, 803) = 2.83, *p* = 0.059, *η*_*p*_^2^ = 0.01), article and quiz condition (*F*(2, 803) = 1.21, *p* = 0.300, *η*_*p*_^2^ = 0.003), and the three-way interaction between article, quiz, and delay conditions (*F*(2, 803) = 0.85, *p* = 0.426, *η*_*p*_^2^ = 0.002) were not significant.

#### Accuracy ratings

**Fig. 2:**
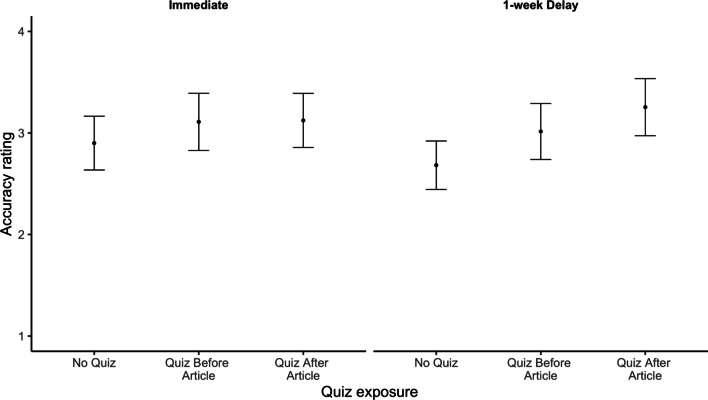
Accuracy ratings for claims by quiz condition and test delay. *Note* Ratings were provided on a 0 (very inaccurate) to 10 (very accurate) scale. Both fact-checked claims were false, so we predicted that quizzes would lower accuracy ratings. Instead, there was no significant effect of quizzing on accuracy ratings. Error bars reflect standard errors of the mean

Finally, we evaluated the effect of quizzing on participants’ evaluation of fact-checked claims. Contrary to our prediction, we did not find that quizzes improved participants’ accuracy ratings (Fig. 2).

We conducted a preregistered 3 (quiz: none, before, after) × 2 (article: DNA, marijuana) × 2 (delay: no delay, delayed) ANOVA on participants’ accuracy ratings. No effects were significant (all *F*s < 3.37). Critically, and contrary to our hypothesis, we did not observe a difference in accuracy ratings between participants who did versus did not receive a quiz (i.e., no main effect of quiz condition, *F*(2, 803) = 1.05, *p* = 0.349, *η*_*p*_^2^ = 0.003).

To follow up on this null result, we conducted an exploratory Bayesian *t* test (using *BayesFactor* version 0.9.12; Morey et al., 2015) comparing accuracy ratings across the quizzed and non-quizzed conditions. Using a default Cauchy distribution with width 0.707, we observed a Bayes factor of 4.59 in favor of the null hypothesis. That is, our data are about 4.59 times more likely under the null hypothesis (that accuracy ratings are identical regardless of quiz status) than under the alternative hypothesis (that there is a difference), constituting moderate evidence for the null (Held & Ott, 2018).

### Discussion

Experiment 1 provided mixed findings about the effects of including multiple-choice quizzes alongside fact checks. Participants answered more quiz questions correctly after reading a fact check than before reading it. Thus, participants did learn from reading the fact check. Including a quiz with a fact check also improved participants’ memory for details from the fact check. The positive effects of quizzing were similar regardless of whether the quiz occurred before or after reading the article. Notably, including a quiz with a fact check did not decrease participants’ accuracy ratings of the false claims that were the subject of the fact check. Overall, quizzes improved memory for details within the fact check but were ineffective at further reducing belief in misinformation.

One possible explanation for the null effects of quizzing on accuracy ratings might be the content of the quiz questions themselves. In Experiment 1, the questions referred to detailed information from the fact check such as “What is the blood–brain barrier?” or “What is a double-blind placebo study?” However, the questions were not directly related to why the fact-checked claim was false. Experiment 2 builds on Experiment 1 by using multiple-choice questions that refer more directly to the fact-checked false claim. We expected that changing the focus of the quiz questions would improve participants’ accuracy in assessing the false claim while retaining the positive effects of quizzing on memory we observed in Experiment 1. To simplify the design, we also eliminated the quiz-before condition (given that both quizzes were equally effective) and removed the quizzed/not-quizzed manipulation on the final test (given that quizzes only benefitted memory for the quizzed items).

## Experiment 2

### Method

Experiment 2 explores how including a quiz that is more targeted to the false claim influences individuals’ memory for information in the article and their accuracy rating of the false claim. We used a 2 (quiz condition: quiz, no quiz) × 2 (article condition: DNA, marijuana) × 2 (delay: no delay, 1-week delay) between-subjects design and again conducted the experiment online using Qualtrics. Data were collected from August 24 to September 3, 2020.

#### Participants

Participants were again recruited using the CloudResearch platform (*N* = 607). Our preregistered sample size (*N* = 600) was chosen to have 75 participants assigned to each condition. The additional participants were again due to automatic oversampling in mTurk. US residents aged 18 years or older were eligible to participate. Participants were compensated $1.21 for their participation in the initial survey and $0.25 for their participation in the survey 1 week later. As preregistered, participants who were assigned to receive the delayed post-treatment questionnaire but did not respond were excluded from the data set (*N* = 77). All participants answered both cued-recall questions. Thus, we arrived at a final sample of *N* = 530. A sensitivity analysis in G*Power indicated that this final sample size had 80% power to detect a between-subjects main effect of quiz condition (quiz, no quiz) on accuracy ratings of at least *f* = 0.12.

The demographic makeup of the sample was primarily White (81.1%) and male (52.2%). Average age was 42.4 years old (SD = 12.1). Average time spent on the self-paced immediate survey was 8 min and 13 s. Average time spent on the self-paced delayed survey was 7 min and 15 s.

#### Materials

The fact checks were identical to Experiment 1. However, we changed the wording of the quiz questions to relate more closely to the claim being fact-checked. For each article, we created two multiple-choice quiz questions about details directly related to the primary claim of the fact check. These questions targeted details that were inconsistent with the key false claim, or details that undermined the “evidence” that people online had used to support the claim. For example, for an article debunking the false claim that “Women Retain DNA from Every Man They Have Ever Slept With,” one question is: “Scientists have speculated that male cells may enter the female brain after intercourse. However, they say it is highly unlikely that: (a) The male and female cells are genetically distinct (b) the male cells could cross the blood–brain barrier (c) pregnancy would lead to more male cells than intercourse (d) this process would happen with every sexual partner” (correct answer: d). As in Experiment 1, each multiple-choice question was turned into a cued-recall question by removing the answer choices (e.g., “Scientists have speculated that male cells may enter the female brain after intercourse. However, they say it is highly unlikely that:”). To measure participants’ belief in the false claim, they were again asked to rate the accuracy of the debunked statement, using the same response options as in Experiment 1. Again, because the claims were entirely false, we expected participant accuracy ratings to be lower on this scale when they were in the quizzed condition.

The full materials are available at https://osf.io/rfchq/?view_only=0185e2f8c3a343cea3bff597dde2f979.

#### Procedure

The procedure was identical to Experiment 1 except that we removed the quiz-before condition. Participants either read the fact-checking article by itself or read the article and then answered two multiple-choice quiz questions with feedback. Participants in the immediate test condition then answered two cued-recall questions and rated the accuracy of the fact-checked claim. One week later, participants in the delayed test condition answered the same two cued-recall questions and provided an accuracy rating.

### Results

All data are available at the online supplement, along with preregistration of our analyses, hypotheses, and sample size: https://osf.io/rfchq/?view_only=0185e2f8c3a343cea3bff597dde2f979.

#### Multiple-choice quizzes

First, we present participants’ accuracy on the multiple-choice quizzes. The average proportion of correct responses to quiz questions across participants was 0.71 (SD = 0.32). Participants who read the marijuana article answered a numerically greater proportion of quiz questions correctly (*M* = 0.75, SD = 0.29) than those who read the DNA article (*M* = 0.67, SD = 0.35), though this difference was not significant, (*t*(271) = − 1.88, *p* = 0.061, *d* = 0.23).

#### Cued-recall questions

**Fig. 3:**
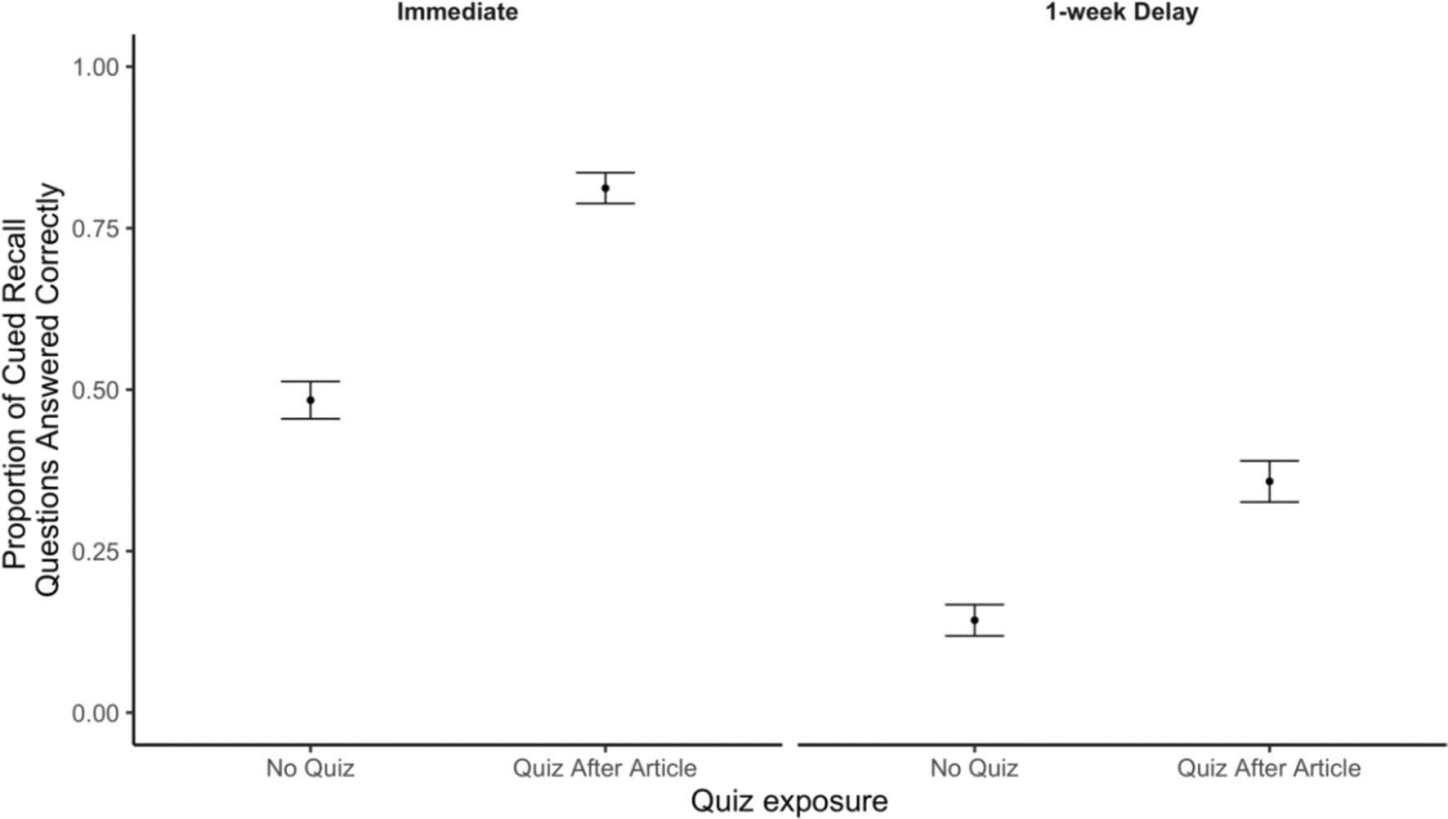
Proportion of cued-recall questions answered correctly by quiz condition and test delay. *Note* Error bars reflect standard errors of the mean

Next, we examined participants’ responses to the open-end cued-recall questions. Recall that participants answered two detailed questions per article. Responses were again scored as correct or incorrect by two independent research assistants (Krippendorff’s alpha = 0.91; all discrepancies were resolved by a co-first author). As in Experiment 1, multiple-choice quizzes improved memory for key details as to why the claims were false (Fig. 3).

We examined the differences in participants’ memory for key details from the fact checks by conducting a preregistered 2 (quiz: quiz, no quiz) × 2 (article: DNA, marijuana) × 2 (delay: no delay, 1-week delay) ANOVA on participants’ proportion of correct responses. We again observed a significant main effect of quizzing (*F*(1, 522) = 93.12, *p* < 0.001, *η*_*p*_^2^ = 0.15). Participants who were quizzed (*M* = 0.61) answered a greater proportion of open-ended questions correctly than participants who were not quizzed (*M* = 0.34). We also observed a significant main effect of article (*F*(1, 522) = 5.86, *p* = 0.016,* η*_*p*_^2^ = 0.01) with participants who saw the marijuana fact check (*M* = 0.53) answering more questions correctly than participants who saw the DNA fact check (*M* = 0.43). There was also a significant main effect of delay (*F*(1, 522) = 196.89, *p* < 0.001, *η*_*p*_^2^ = 0.27). Participants who answered questions immediately after reading the article were more accurate (*M* = 0.65) than participants who answered 1 week later (*M* = 0.26). Additionally, we observed a significant interaction between article and delay, (*F*(1, 522) = 13.13, *p* < 0.001, *η*_*p*_^2^ = 0.03; the effect of delay was stronger for the marijuana article), a significant interaction of quiz condition and delay, (*F*(1, 522) = 4.15, *p* = 0.043, *η*_*p*_^2^ = 0.01; the benefit of quizzing was larger on the immediate test), and a significant interaction of quiz condition and article, (*F*(1,522) = 3.90, *p* = 0.049, *η*_*p*_^2^ = 0.01; the effects of quizzing were larger for the DNA article). The three-way interaction of quiz condition, article, and delay was not significant (*F* < 1).

#### Accuracy ratings

**Fig. 4:**
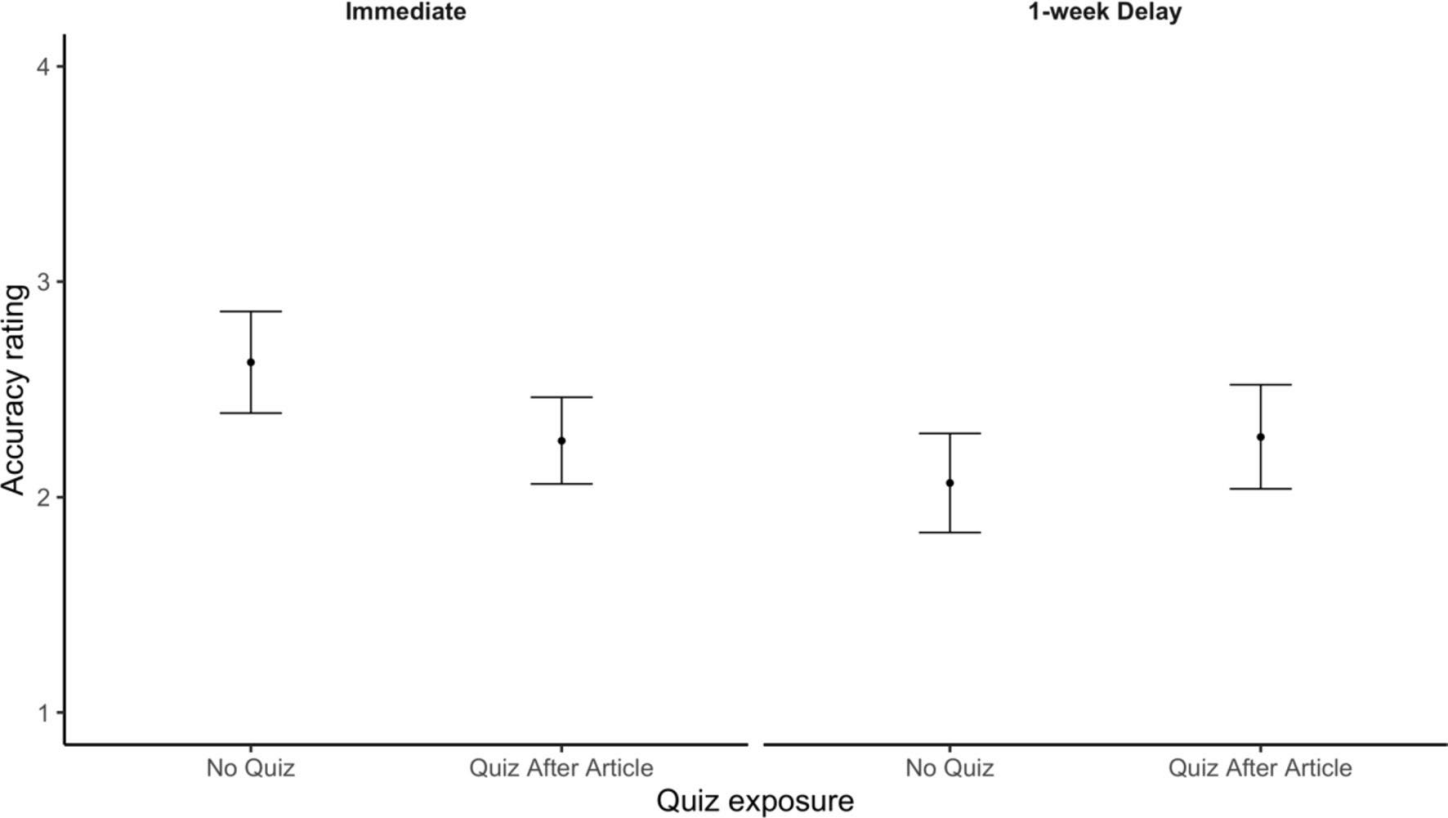
Accuracy ratings for false claims by quiz condition and test delay. *Note* Ratings were provided on a 0 (very inaccurate) to 10 (very accurate) scale. Error bars reflect standard errors of the mean

Next, we evaluated the effect of quizzing on participants’ evaluation of the fact-checked claims. Our key question was whether quizzes would decrease participants’ accuracy ratings for the false claims. We again found that quizzes did not improve accuracy ratings (Fig. 4). As a reminder, the claims used in Experiment 2 were rated false, so we expected participants’ ratings to be *lower* after reading the fact checks and receiving a quiz.

We conducted a preregistered 2 (quiz: none, after) × 2 (article: DNA, marijuana) × 2 (delay: no delay, 1-week delay) ANOVA on participants’ mean accuracy ratings. We observed a significant main effect of the article condition on accuracy (*F*(1, 522) = 4.96, *p* = 0.026, *η*_*p*_^2^ = 0.01) where participants who read the marijuana fact check (*M* = 2.58) rated the claim as more accurate than participants who read the DNA fact check (*M* = 2.07). No other effects were significant (all *Fs* < 2.67). Critically, we again did not see a main effect of quizzing (*F* = 0.18, *p* = 0.668, *η*_*p*_^2^ < 0.01). Overall, the results do not suggest that quizzes improve participants’ accuracy ratings for fact-checked claims.

As in Experiment 1, we followed up on this null result by conducting a Bayesian *t* test comparing accuracy ratings for claims across quizzed and non-quizzed conditions. Again, we observe moderate evidence in favor of the null hypothesis that accuracy ratings are the same for participants in the quizzed and non-quizzed conditions: The Bayes factor for this test was 8.78 in favor of the null.

### Discussion

The results of Experiment 2 again suggest that multiple-choice quizzes improve participants’ memory for key details from fact checks (both immediately and 1 week later), but they do not decrease participants’ belief in the false claims. In Experiment 3, we again examined the effects of multiple-choice quizzes on memory and belief, while remedying four limitations from the previous studies. First, Experiments 1 and 2 measured belief using a standard 11-point accuracy scale. However, this type of scale may fail to capture the degree to which participants believe or disbelieve a claim. Experiment 3 incorporated the prior measure of accuracy as well as a second open-end measure to assess both belief and disbelief of fact-checked claims.

Second, a possible explanation for the null accuracy findings in Experiments 1 and 2 is that while we did improve memory for details from the fact checks, participants did not need to use that memory to decide that the fact-checked claim was false. In both experiments, participants saw a single fact check that assessed a single claim as false. Therefore, when participants reached the accuracy rating, they could likely infer that the claim they saw during the experiment was false if they generally remembered seeing a similar claim in the fact check. To fix this limitation, in Experiment 3, we showed participants multiple articles that fact-checked both true and false claims. In this situation, participants will need to remember not only if they saw a claim but also if the article declared it to be true or false, providing a stronger test of our hypothesis.

Third, an alternate possibility for our null findings is that the articles themselves were not effective and could not shift accuracy ratings, regardless of whether a quiz was present. While past work suggests this is not likely and that fact checks are generally effective (Walter et al., 2020), we are unable to rule out this possibility because our previous experiments did not have a control condition. Thus, in Experiment 3, we added a no-exposure control, where participants answered cued-recall questions and provided accuracy and belief measures for articles they did not read. Finally, Experiment 3 used fact checks of true and false political claims to extend our findings beyond health-related topics.

## Experiment 3

### Method

Experiment 3 explores how including a multiple-choice quiz with true and false fact-checked claims influences individuals’ accuracy rating of the claim, belief in the claim, and memory for key details in the article related to whether claims were true or false. To address our hypotheses, we conducted an online survey experiment administered through Qualtrics. The truth of each fact-checked claim (true, false) and quiz condition (no quiz, quiz) varied within-subjects along with exposure to the fact check (exposed, not exposed). For Experiment 3, there was no immediate test; all participants answered the memory and belief questions 1 week after reading the articles. Data were collected between September 2 and 10, 2021.

#### Participants

Participants were again recruited using CloudResearch (*N* = 300). The preregistered sample size was chosen to have 300 participants assigned to each within-subjects condition, which would allow us to detect smaller effects that might not have been observed in Experiments 1 and 2 as well as account for attrition among those who did not complete the post-treatment survey 1 week later. US residents aged 18 years or older were eligible to participate. Participants were compensated $1.81 for their participation in each session. As preregistered, participants who did not complete the delayed post-treatment questionnaire were excluded from the dataset (*N* = 94). All participants answered all the cued-recall and belief measures. Thus, we arrived at a final sample of 206 participants. A sensitivity analysis using SuperPower (Lakens & Caldwell, 2021) revealed that this sample size had 80% power to detect an interaction effect between quizzing and truth on accuracy ratings such that quizzes increased ratings for true claims and decreased ratings for false claims by 0.6 points relative to the no-quiz condition (corresponding to effect size of *η*_*p*_^2^ = 0.04). This power analysis assumed a standard deviation of 3.4, a correlation among repeated measures of 0.23, and mean accuracy ratings in the no-quiz condition of 6.53 (true items) and 3.09 (false items), as observed in this experiment.

The demographic makeup of the sample was primarily White (80.5%) and male (52.1%). Average age was 40.38 years (SD = 10.76). Average time spent on the self-paced initial survey was 14 min 53 s. Average time spent on the self-paced delayed survey was 14 min 59 s.

#### Materials

**Fig. 5:**
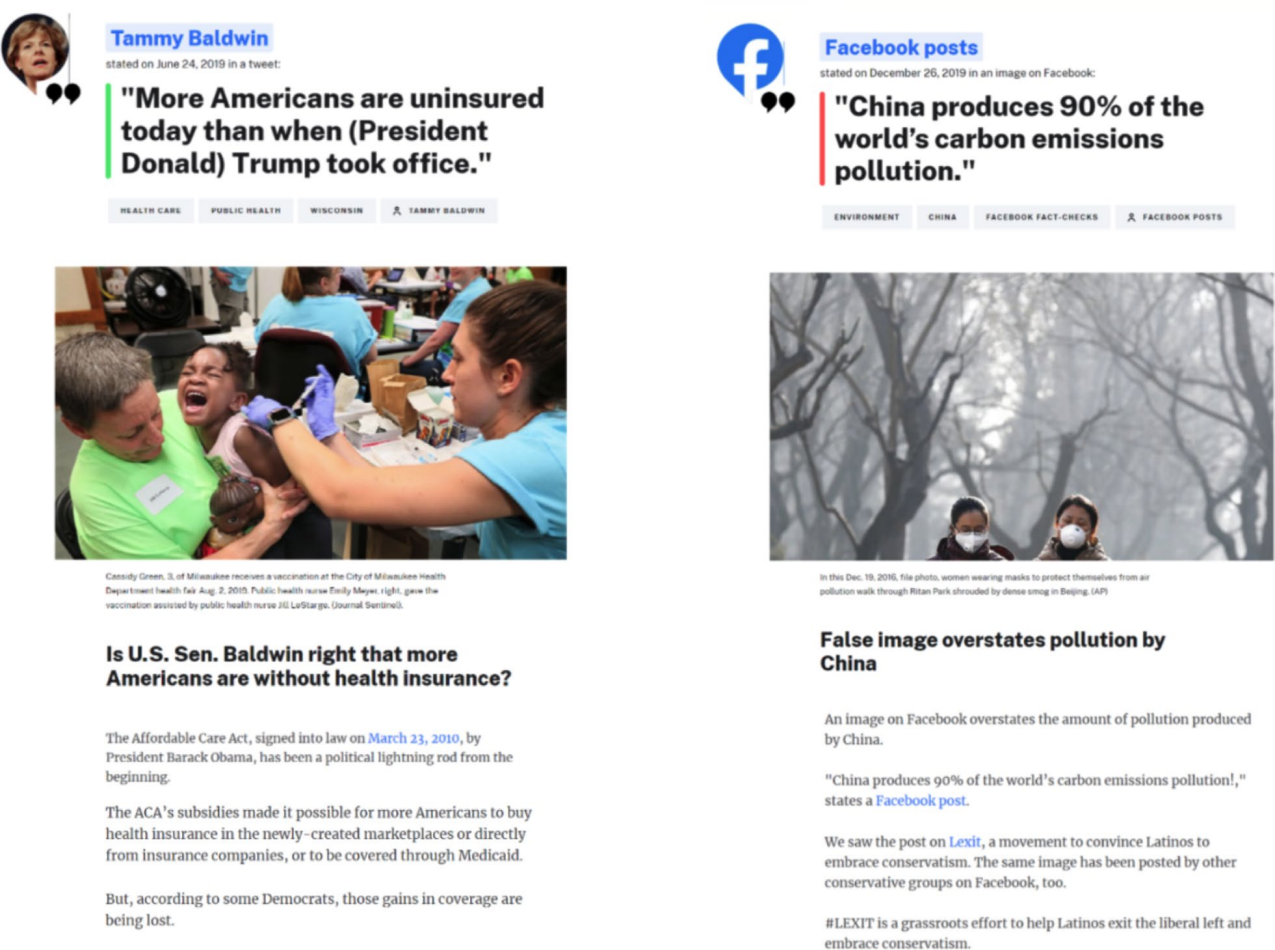
Sample screenshots of the top of an article about a true (left) and false (right) claim

We tested the efficacy of the quiz intervention across eight different articles selected from a well-known fact-checking organization for US politics and politicians, PolitiFact (Table 2). Claims were balanced across partisanship such that four articles were favorable to Democrats and four were favorable to Republicans. Additionally, four claims were rated true and four were rated false by PolitiFact (split evenly across partisanship). All articles were presented to participants without any sponsored content (e.g., advertisements, links to other articles on PolitiFact). In addition, while PolitiFact articles typically begin with a “Truth-o-Meter” graphic depicting how true or false the fact-checked claim was, we removed this graphic prior to presenting the articles to participants. Sample screenshots of two articles as they were presented to participants are shown in Fig. 5, and full screenshots are available at the project’s OSF site.

**Table 2:** Claims and truth of claims used in study 3 stimuli

Claim	True/false
China produces 90% of the world’s carbon emissions pollution	False
As of 2019, the number of murders in most American cities besides Chicago had dropped by over 10% in the prior 2 years	False
Bernie Sanders’ Medicare for all plan would place a 52% tax on earnings over $29,000	False
Half of those arrested for DUIs on I-35 are in the country illegally	False
More Americans were uninsured in 2019 than when President Donald Trump took office	True
The US has the lowest food stamp rolls in years, as of February 2019	True
Communities along the Texas–Mexico border have seen a 25% reduction in crime from 2014 to 2018	True
The United States has more governors who have worn blackface than Black governors	True

From these eight articles, we created two sets of four articles to counterbalance which set was shown to each participant and which set of items were “not read.” Each set contained fact checks about two true claims and two false claims. Within each set of fact checks, we created two pairs, each containing a fact check of one true and one false claim. To determine the order of the fact checks, we randomized the order of the two pairs, and of items within each pair of fact checks. This was done so that quizzes could be placed on the first two or last two articles shown to participants, while ensuring that one true/false item was quizzed and the other was not quizzed.

Since all participants answered the questions 1 week after reading the fact-checking article, they received this instruction before proceeding to the questions:

Last week you read a series of articles from a fact-checking organization. Now, we’d like you to answer a few questions about those articles. Please don’t attempt to look up the answers. Give us your best guess, and if you don’t know the answer, simply write ‘don’t know’.

For each article, we created two multiple-choice questions about details within the article (e.g., “For 2018, the vast majority of countries produced less than __% of the world’s carbon emissions. (a) 10 (b) 25 (c) 5 (d) 1”). As in Experiment 2, the details were closely related to why the fact-checked claim was true or false. Each multiple-choice question was again turned into a cued-recall question by removing the answer choices (e.g., “For 2018, the vast majority of countries produced less than __% of the world’s carbon emissions.”). Accuracy ratings for the fact-checked claims (e.g., “China produces 90% of the world’s carbon emissions pollution.”) were provided on the same 11-point scale from *very inaccurate* (0) to *very accurate* (10). Participants’ belief in the fact-checked claim was also assessed using an open-end measure (e.g., “What do you believe about China producing 90% of the world’s carbon emissions pollution?”). The open-end measure differs from the accuracy ratings as responses could be coded as (1) belief, (2) disbelief, (3) ambivalence/don’t care, (4) don’t know, or (5) opinion or comment. Past research has found that this open-ended measure provides important insights not captured by simple accuracy ratings (e.g., Collier, 2021).

#### Procedure

Participants were first shown four fact checks from PolitiFact, consisting of two fact checks of true claims and two fact checks of false claims. For each fact check, participants either read the article and received a brief, two-question quiz about the information in the fact check or received no quiz at all. These quizzes were randomly placed on either the first two or last two articles read by each participant, and each participant received a quiz for one true claim and one false claim. After reading all four articles, participants completed a basic demographic questionnaire and were compensated for their participation.

One week later, participants were asked to rate the accuracy of the claim for each fact check, answer two cued-recall questions about the content of the fact check, and answer an open-end question about their belief in the fact-checked claim. (Unlike Experiments 1 and 2, there was no immediate test condition.) Participants provided responses for all four measures in the same order (accuracy rating, two cued-recall questions, open-end belief measure) for all eight articles (four read, four not read). The order of the articles varied randomly across participants.

### Results

All data are available at the online supplement, along with preregistration of our analyses, hypotheses, and sample size: https://osf.io/rfchq/?view_only=0185e2f8c3a343cea3bff597dde2f979.

#### Multiple-choice quizzes

First, we present participants’ accuracy on the multiple-choice quizzes. The average proportion of correct responses across participants was 0.59 (SD = 0.29). Average accuracy was lowest for the article regarding the Texas–Mexico border (*M* = 0.42) and highest for the article regarding governors wearing blackface (*M* = 0.79).

#### Cued-recall questions

**Fig. 6:**
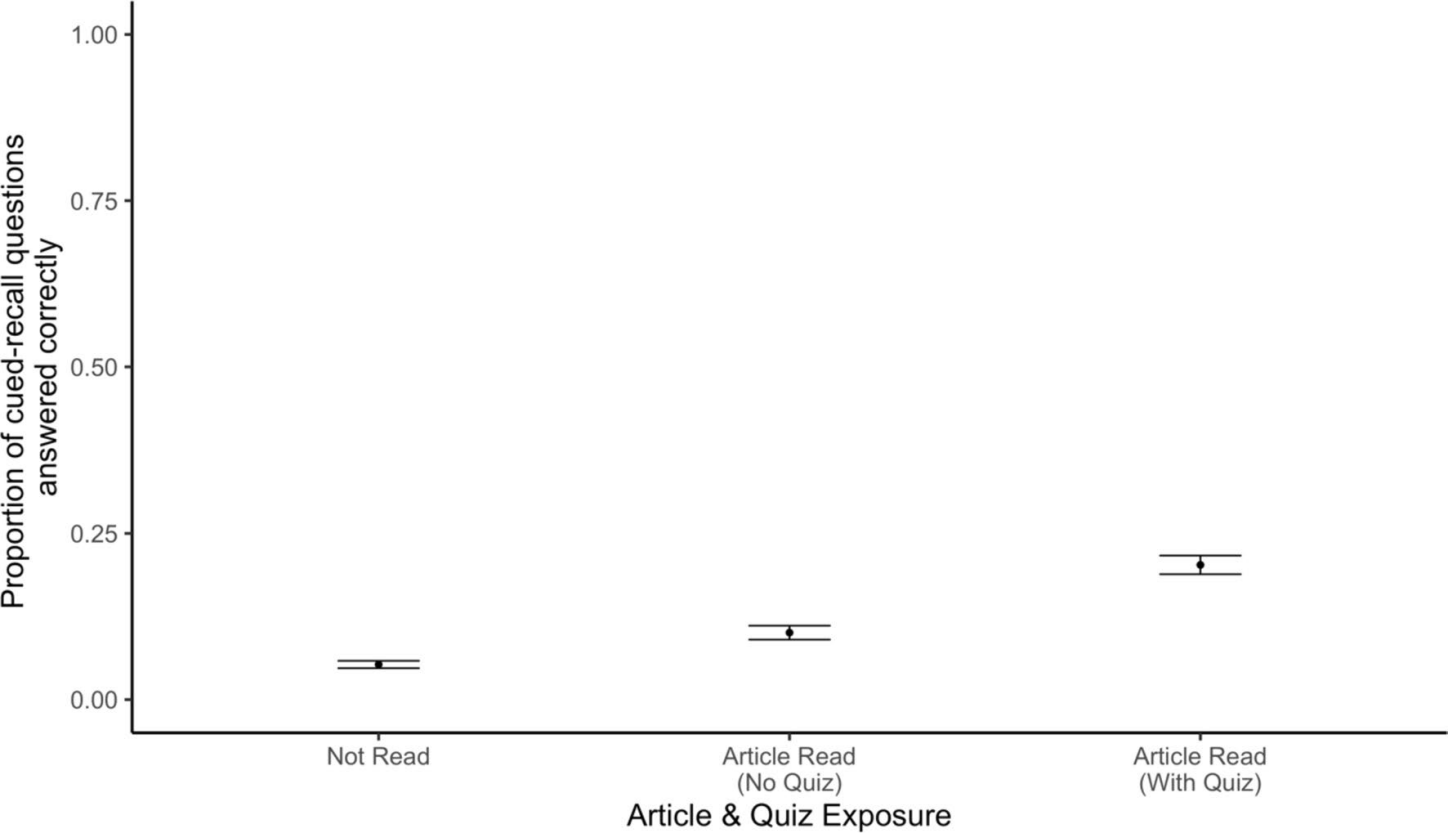
Proportion of cued-recall questions answered correctly by article exposure and quiz condition. *Note* Error bars reflect standard errors of the mean

Next, we examined participants’ responses to the open-ended questions. Recall that participants answered two detailed questions about each claim/article, and that scored responses were coded as either correct or incorrect. As in the previous experiments, responses were scored by two independent coders (Krippendorff’s alpha = 0.72) with all discrepancies resolved by a co-first author. Our key question was whether quizzing would affect memory for the details from the article. As shown in Fig. 6, it did. Participants answered more cued-recall questions correctly, on average, for articles that were quizzed relative to articles that were not quizzed.

To evaluate these data statistically, we conducted a preregistered 2 (quiz condition: no quiz, quiz) × 2 (claim truth: true, false) ANOVA on proportion of cued-recall questions answered correctly. The analysis only included the subset of questions corresponding to read articles (eight questions). We observed a significant main effect of quiz condition (*F*(1,205) = 33.63, *p* < 0.001, *η*_*p*_^2^ = 0.15) such that accuracy on the questions was greater when participants were originally quizzed (*M* = 0.20) than when they were not quizzed (*M* = 0.10). We did not observe a significant effect of claim truth (*F* < 1) or an interaction between claim truth and quiz condition (*F*(1, 205) = 1.58, *p* = 0.211, *η*_*p*_^2^ = 0.01). Overall, quizzes improved memory for details of both articles that debunked false claims and those that affirmed true claims.

#### Accuracy ratings

**Fig. 7:**
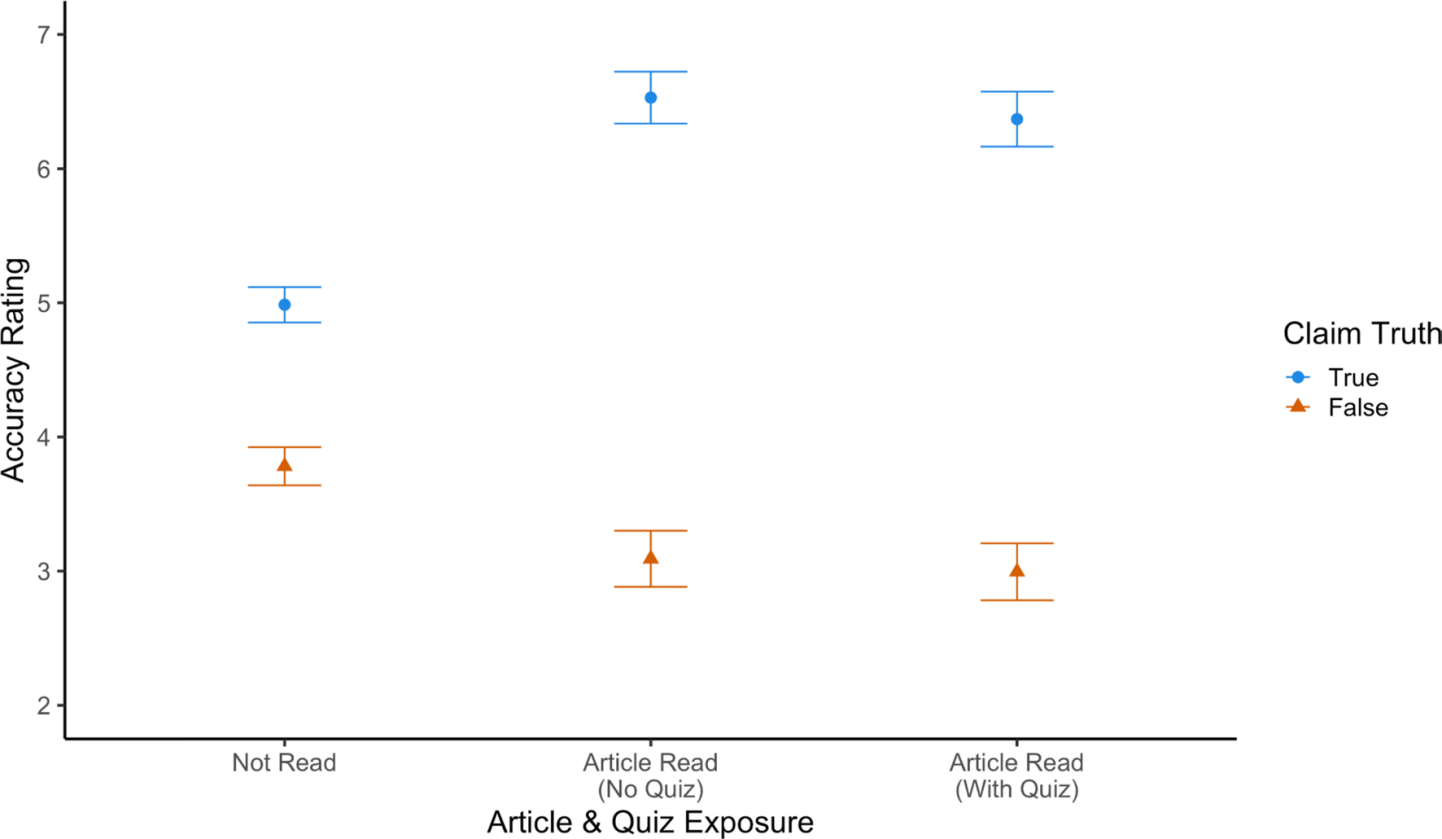
Accuracy ratings for claims by claim truth, article exposure and quiz condition. *Note* Ratings were provided on a 0 (very inaccurate) to 10 (very accurate) scale. Error bars reflect standard errors of the mean

Next, we examined participants’ accuracy ratings for the true and false claims. Our first question was whether fact checks were effective at changing the accuracy of people’s evaluations of true and false claims. As shown in Fig. 7, they were. When participants read the corresponding articles, they gave higher accuracy ratings to true claims and lower accuracy ratings to false claims, relative to when they had not read the articles. Our second question was whether quizzing provided any additional benefit above and beyond simply reading the fact check. As shown in Fig. 7, this was not the case. Ratings were similar for articles that were encountered with and without a quiz.

To evaluate the effect of exposure to fact-checking articles statistically, we conducted a preregistered 2 (claim truth: true, false) × 2 (article exposure: read, not read) ANOVA on participants’ mean accuracy ratings. We observed a significant main effect of claim truth (*F*(1,205) = 186.76, *p* < 0.001, *η*_*p*_^2^ = 0.48) such that participants gave higher ratings to true claims (*M* = 5.72) than false claims (*M* = 3.41) on average. We also observed a significant main effect of article exposure (*F*(1,205) = 6.07, *p* = 0.015, *η*_*p*_^2^ = 0.03), such that participants gave higher ratings on average to claims when they had read a fact check (*M* = 4.75) than when they had not (*M* = 4.38). Critically, these main effects were qualified by a significant interaction between claim truth and article exposure (*F*(1,205) = 69.38, *p* < 0.001, *η*_*p*_^2^ = 0.25). Consistent with our hypothesis, exposure to fact checks was effective at increasing accuracy ratings of true claims (M_not read_ = 4.99, M_read_ = 6.45, *t*(205) = 8.03, *p* < 0.001, *d* = 0.56) while decreasing ratings for false claims (M_not read_ = 3.78, M_read_ = 3.04, *t*(205) = − 3.48, *p* < 0.001, *d* = 0.24).

Next, to evaluate the effects of quizzes on accuracy ratings, we conducted a preregistered 2 (quiz condition: quiz, no quiz) × 2 (claim truth: true, false) ANOVA on participants’ accuracy ratings for the subset of claims for which they read fact checks. Consistent with the results reported above, we observed a significant main effect of claim truth (*F*(1,205) = 240.01, *p* < 0.001, *η*_*p*_^2^ = 0.54) such that participants gave higher ratings to true claims (*M* = 6.45) than to false claims (*M* = 3.04). However, we did not observe a significant main effect of quiz condition, nor a significant interaction between quiz condition and claim truth (*F*s < 1). Thus, contrary to our hypothesis, we did not observe evidence that quizzing affected accuracy ratings.

As in Experiments 1 and 2, we again followed up on these null results by conducting Bayesian *t* tests comparing accuracy ratings for quizzed versus non-quizzed items. Note that we here used a matched-pairs Bayesian *t* test, as, unlike in Experiments 1 and 2, quiz status was manipulated within-subjects. In addition, given that our hypothesis predicted that quizzing would have opposite effects on articles about true and false claims (i.e., that quizzes would increase accuracy ratings of true claims and decrease ratings for false claims), we analyzed these data in two separate *t* tests. Overall, we find strong evidence in favor of the null hypothesis that accuracy ratings were the same regardless of whether participants were or were not quizzed after originally reading the article; the Bayes factor was 10.78 in favor of the null for true items and 11.82 in favor of the null for false items.

#### Belief in claims

**Fig. 8:**
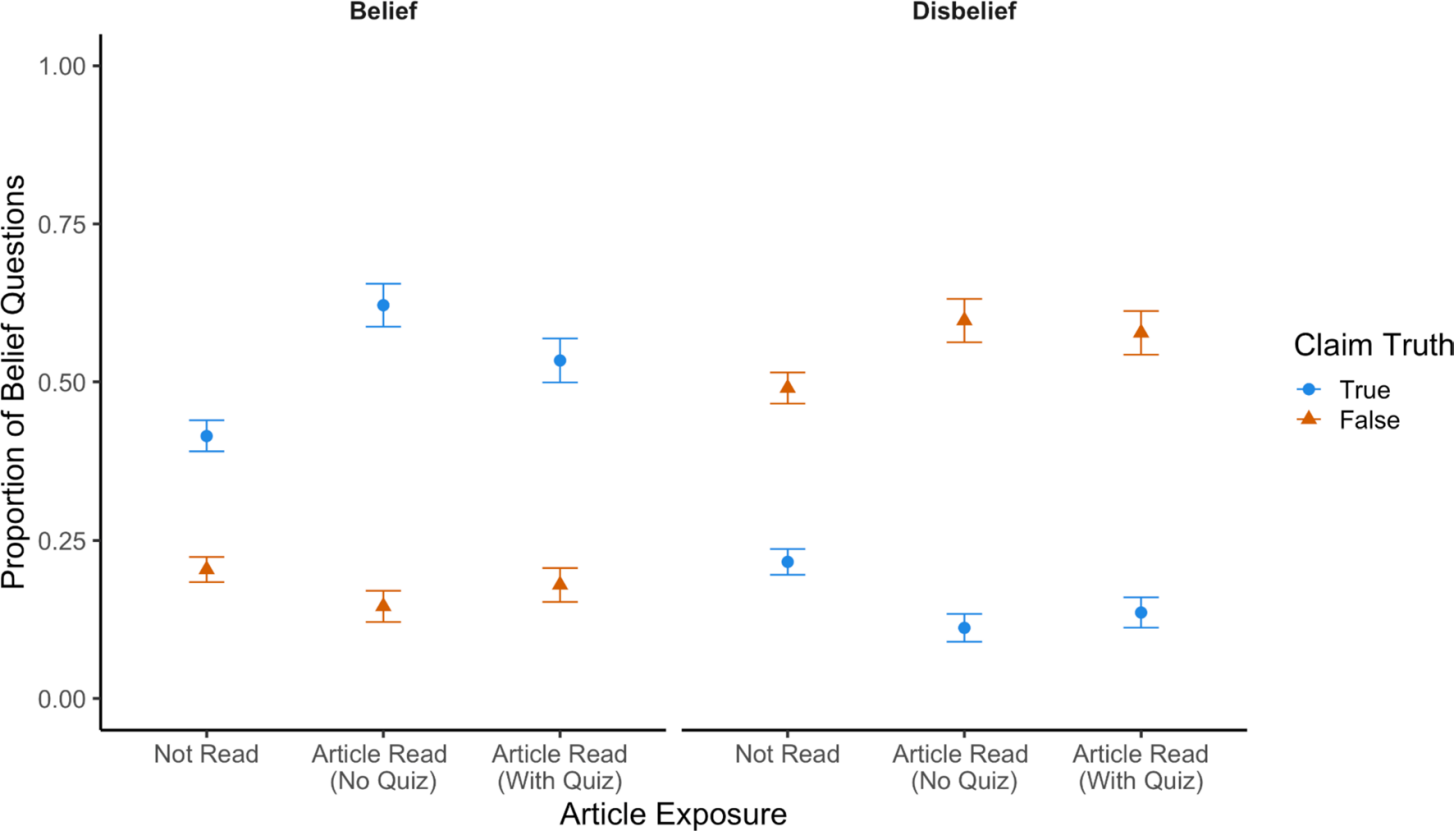
Proportion of belief questions coded as belief or disbelief, by claim truth, article exposure, and quiz condition. *Note*. Left panel reflects the proportion of answers scored as “belief.” Right panel reflects the proportion of answers scored as “disbelief.” Answers may also have been scored as ambivalence/don’t care, don’t know, or opinion/comment. Error bars reflect standard errors of the mean

Finally, we examined participants’ open-ended responses to a self-report measure of belief. Answers were scored as (1) belief, (2) disbelief, (3) ambivalence/don’t care, (4) don’t know, or (5) opinion or comment by two independent research assistants (Krippendorff’s alpha = 0.43). While reliability was lower than expected, all discrepancies were resolved by a co-first author prior to analysis. Responses were mostly categorized as belief (33.98%) or disbelief (35.44%), with lower rates for ambivalent (0.97%), don’t know (9.34%), or opinion (20.27%) responses. Figure 7 shows the proportion of responses that were scored as belief (left panel) and disbelief (right panel), by claim truth, article exposure, and quiz condition. Our first question was whether exposure to fact checks would improve the accuracy of peoples’ beliefs on this measure. As shown in the left panel of Fig. 8, it did. Participants believed true claims more often than false claims, and this difference was greater for claims relating to articles that participants had read. Our second question was whether quizzes affected this process of belief change. We did not see strong evidence of this. As shown in both panels, participants believed and disbelieved claims to similar extents, regardless of whether they had read the original article with or without a quiz.

To evaluate these data statistically, we first conducted a preregistered 2 (claim truth: true, false) × 2 (article exposure: read, not read) ANOVA on the mean proportion of responses scored as “belief.” We observed a significant main effect of claim truth (*F*(1,205) = 165.24, *p* < 0.001, *η*_*p*_^2^ = 0.45) such that participants believed true claims (*M* = 0.50) more often than false claims (*M* = 0.18). We also observed a significant main effect of article exposure (*F*(1,205) = 8.89, *p* = 0.003, *η*_*p*_^2^ = 0.04), such that participants believed claims about which they had read a fact check (*M* = 0.37) more often than when they had not (*M* = 0.31). Critically, these main effects were qualified by a significant interaction between claim truth and article exposure (*F*(1,205) = 22.67, *p* < 0.001, *η*_*p*_^2^ = 0.10). Exposure to fact checks increased belief in true claims (M_not read_ = 0.42, M_read_ = 0.58, *t*(205) = 5.11, *p* < 0.001, *d* = 0.36), but we did not observe a significant change in belief for false claims (M_not read_ = 0.20, M_read_ = 0.16, *t*(205) = − 1.53, *p* = 0.129, *d* = 0.11).

In an exploratory follow-up analysis, we again conducted a 2 (claim truth: true, false) × 2 (article exposure: read, not read) ANOVA, this time on the proportion of responses scored as “disbelief.” Note that “belief” and “disbelief” are not the only two possible responses; items may also have been rated “don’t know,” “don’t care,” or “opinion.” We observed significant main effect of claim truth (*F*(1,205) = 265.75, *p* < 0.001, *η*_*p*_^2^ = 0.56) such that participants disbelieved false claims (*M* = 0.54) more often than true claims (*M* = 0.17). This main effect was qualified by a significant interaction between claim truth and article exposure (*F*(1,205) = 18.55, *p* < 0.001, *η*_*p*_^2^ = 0.08). Exposure to fact checks increased disbelief in false claims (M_not read_ = 0.49, M_read_ = 0.58, *t*(205) = 2.89, *p* = 0.004, *d* = 0.20) and decreased disbelief in true claims (M_not read_ = 0.21, M_read_ = 0.12, *t*(205) = − 1.53, *p* < 0.001, *d* = 0.25). We did not observe a significant main effect of article exposure (*F* < 1).

Next, we examined the effects of quizzing on belief. Note that participants saw only four articles. Accordingly, each participant had only one belief measure observation for each combination of claim truth (true, false) and quiz condition (quiz, no quiz). Thus, instead of using an ANOVA on the proportion of answers given a certain score, we used a McNemar’s test. This allowed us to account for the nominal nature of our data, as well as the fact that our independent variables were manipulated within-subjects.

To examine the effects of quizzing on belief, we first conducted a preregistered McNemar’s test on the number of true claims that were believed by participants, split by whether they did or did not take a quiz after reading the corresponding article. We did not observe a significant effect of quiz condition on participant’s belief in true claims (*χ*^*2*^(1) = 3.44, *p* = 0.064). Note that the effect was both not significant, and it was in the opposite direction as predicted (i.e., the mean proportion of responses scored as belief was *lower* after quizzing). Next, we conducted an identical preregistered McNemar’s test, but for false claims that were disbelieved by participants. We did not observe a significant effect of quiz condition (*χ*^*2*^(1) = 0.11, *p* = 0.743) on disbelief in false claims. Overall, quizzing did not affect participants’ belief in these claims.^3^

### Discussion

Despite incorporating additional contexts and measures in Experiment 3, results were largely consistent with Experiments 1 and 2. Multiple-choice quizzes improved participants’ memory for details from fact checks but did not improve participants’ accuracy ratings. Participants answered more questions correctly for articles that were quizzed compared to articles that were not accompanied by a quiz. We observed this effect of quizzing across both true and false claims. While participants’ accuracy ratings were higher for true claims than false claims, quizzing did not significantly affect accuracy ratings. Similar results were found with our new open-ended measure of belief. While participants reported believing true claims more than false claims, multiple-choice quizzes did not influence belief or disbelief in the fact-checked claims. It is important to note that this new open-ended belief measure is that reliability was low (Krippendorff’s alpha = 0.43), perhaps due to the complexity of the coding scheme. While a co-first author resolved all discrepancies prior to analysis, it is worth noting the low reliability as a caveat when interpreting these findings. Finally, across measures, exposure to fact checks in general improved participants’ accuracy ratings and belief/disbelief in both true and false claims.

## General discussion

First and foremost, the results from this study support previous findings that exposure to fact checks reduces belief in false claims (see Walter & Murphy, 2018 for a review). In Experiment 3, participants who read a fact check were more accurate in their ratings of true and false claims compared to those who did not read a fact check. Regarding multiple-choice quizzes, we find across three experiments that quizzes do not boost the effect of reading fact checks on belief. Across all three experiments, we consistently find moderate to strong Bayesian evidence in favor of this null effect. Still, quizzes do improve memory for key details from within the fact checks. It is unclear why multiple-choice quizzes may increase memory for fact check details while not affecting belief in the fact-checked claims. As mentioned before, fact checks are generally more effective when they contain more details (e.g., Ecker et al., 2020b), so one might expect manipulations that support memory for such details to decrease belief in false claims. Here, we present three possible explanations for our pattern of findings.

First, while quizzing boosted memory for information presented in the article, it may not have made participants more likely to retrieve or use this information when reporting their belief in the fact-checked claim. That is, the boost in memory due to quizzing was apparent on the cued-recall questions that directly assessed memory for article details, but not on the accuracy rating or open-ended belief measures, which only indirectly rely upon this memory. This explanation is consistent with a broader phenomenon of *knowledge neglect*, in which people fail to use stored knowledge in relevant contexts (see Marsh & Umanath, 2013 for a review). Retrieving knowledge is effortful, so people may fail to recognize contradictions between their knowledge and errors found in fictional stories (Fazio et al., 2013) or in the premises of questions (Erickson & Mattson, 1981). Similarly, people may fail to retrieve information about fact check details when evaluating the truth of fact-checked claims, even if that information was strengthened in memory through quizzing. One flaw for this explanation is that if people tend to ignore their existing knowledge while judging claims, it does not explain why exposure to fact checks themselves improves the accuracy of peoples’ beliefs. One possibility is that there is some minimal level of memory for a relevant fact check that may affect beliefs, after which enhancing memory is unlikely to have an impact.

Second, the benefits of quizzing people about details may not transfer to retention of higher-order information, like the key argument of an article. For instance, retrieval practice with lower-order information from Bloom’s taxonomy (e.g., recognizing or recalling details from the passage) does not enhance performance on questions tapping higher levels (e.g., evaluating an author’s argument, applying content to a new context) (Agarwal, 2019). Instead, retrieval practice has more limited, context-dependent benefits, improving performance on similar questions (e.g., lower-order questions after lower-order quizzes and higher-order questions after higher-order quizzes; see discussion of moderators of the effects of testing on transfer of learning in Pan & Rickard, 2018). Similarly, retrieving details from a fact check may not help readers retain the article’s overall argument that a claim is true or false.

Third, judgments about the accuracy of fact-checked claims may not be constructed based on memory for details of the fact check itself. Instead, these judgments may depend on memory for the mental evaluations of the claim people make when they are initially processing the fact checks (Hastie & Park, 1986). It is possible that participants find it sufficiently easy to recall the false tag or remember having judged the claim as inaccurate after reading, and that remembering details from the fact check does not provide further assistance. Thus, corrections may be more effective with manipulations that create stronger initial evaluations as people read the fact check (e.g., increasing the details in fact checks; Ecker et al., 2020a), but not with manipulations that affect memory for fact check details after the fact (e.g., quizzes).

A key limitation of the present work is that we are unable to directly address these possible explanations. Future work is needed to explain why quizzing benefits memory for article details but not beliefs about the accuracy of fact-checked claims. Still, our findings raise an important constraint on theories of belief updating and processing of corrective information: Greater memory for corrective information alone is not sufficient to improve the efficacy of the correction. This distinction between memory and belief mirrors other subtle, yet important distinctions in the literature on correcting misinformation. For instance, misinformation can have lingering effects on attitudes even after reporting acceptance of, or belief in, a correction (Thorson, 2016). Additionally, fact checks can change the accuracy of peoples’ beliefs about misleading claims but not their evaluations of people making the claims (Nyhan et al., 2020). Thus, there are often dissociations between beliefs and attitudes. Similarly, our work highlights a distinction between memory for corrective information and the beliefs that result from encountering corrective information.

In addition to our main findings about the differential effects of quizzing on memory for details from the fact check and belief, our results also addressed two other questions about the effects of quizzing on memory for information from fact checks. First, we find in Experiment 1 that quizzing boosted memory for general information from the fact check not only when the quiz was after the article, but also when it was before the article, in line with recent work (Pan & Sana, 2021). Second, we also show in Experiment 1 that the memorial benefits of quizzing were limited to quizzed items. Past work has shown benefits of testing even for non-tested materials (e.g., Chan, 2010; Chan et al., 2006), so long as the material is related (e.g., semantically similar). Thus, it is possible that our non-tested materials were not related enough to the quiz conditions to receive a benefit from quizzing. One caveat regarding these findings is that we cannot unambiguously attribute changes in memory for information from the fact check to quizzing, as opposed to merely re-encountering correct information in the quiz answers. We did not include a control condition in which participants merely read the correct answers again, as this did not address our main research questions about the memorial and engagement benefits of an interactive online tool. However, future work may benefit from adding such a condition.

Practically, our research contributes to the burgeoning work on what does and does not work to ensure people hold accurate beliefs (e.g., Ecker et al., 2020a, 2020b). The results of the current studies serve as cautionary evidence for fact-checkers or newsrooms who might expend valuable resources to implement quizzes because they might intuitively be expected to promote accurate beliefs. However, we also show that quizzes may be useful if the goal is to boost memory for specific details in a fact check article. Other work has shown that online quizzes can enhance political interest and engagement with news articles (e.g., Masullo Chen et al., 2018; Scacco et al., 2016). Further, multiple-choice quizzes may be useful for fact-checkers to assess the effectiveness of their articles. Overall, multiple-choice quizzes are not a panacea for misinformation but could be another useful tool that fact-checking organizations might implement to combat misinformation. Importantly, while there may be some benefits to presenting online quizzes about the details of fact checks alongside fact checks, we show that improving the accuracy of peoples’ beliefs is not one of them.

## Data Availability

Full data and materials are available in the OSF repository, https://osf.io/rfchq/?view_only=0185e2f8c3a343cea3bff597dde2f979.

## Footnotes

1 Questions were featured in the December 9, 2022, News Quiz. The source articles for each question are as follows: Supreme Court case – https://www.nytimes.com/2022/12/06/briefing/gay-rights-supreme-court.html and Brittany Griner – https://www.nytimes.com/live/2022/12/08/world/brittney-griner-russia-ukraine-news.

2 For Experiments 1 and 2, we also preregistered analyses to test effects for the immediate and delayed conditions separately. For simplicity, we report the full 3 × 2x2 ANOVAs in the main text. All additional analyses not reported here are available in the OSF repository: https://osf.io/rfchq/?view_only=0185e2f8c3a343cea3bff597dde2f979.

3 In exploratory McNemar’s tests, we also observed non-significant effect of quizzing on *disbelief* in true claims (*χ*^*2*^(1) = .63, *p* = .427) and on *belief* in false claims (*χ*^*2*^(1) = .39, *p* = .532).
